# Role of ZnO Nanoparticles Loading in Modifying the Morphological, Optical, and Thermal Properties of Immiscible Polymer (PMMA/PEG) Blends

**DOI:** 10.3390/ma15238453

**Published:** 2022-11-27

**Authors:** Salim Hammani, Sihem Daikhi, Mikhael Bechelany, Ahmed Barhoum

**Affiliations:** 1 Laboratoire Chimie Physique Moléculaire et Macromoléculaire, Université Saad Dahlab Blida1, Route de Soumaa, BP 270, Blida 09000, Algeria; 2 Institut Europeen des Membranes, IEM, UMR 5635, University of Montpellier, ENSCM, CNRS, 34730 Montpellier, France; 3 NanoStruc Research Group, Chemistry Department, Faculty of Science, Helwan University, Cairo 11795, Egypt; 4 School of Chemical Sciences, Dublin City University, Dublin 9, D09 Y074 Dublin, Ireland

**Keywords:** polymer nanocomposites, nanoparticle dispersion, UV blocking, bandgap energy, melting temperature, crystallization temperature, thermal stability

## Abstract

High-performance hybrid polymer blends can be prepared by blending different types of polymers to improve their properties. However, most polymer blends exhibit phase separation after blending. In this study, polymethylmethacrylate/polyethylene glycol (PMMA/PEG) polymer blends (70/30 and 30/70 w/w) were prepared by solution casting with and without ZnO nanoparticles (NPs) loading. The effect of loading ZnO nanoparticles on blend morphology, UV blocking, glass transition, melting, and crystallization were investigated. Without loading ZnO NP, the PMMA/PEG blends showed phase separation, especially the PEG-rich blend. Loading PMMA/PEG blend with ZnO NPs increased the miscibility of the blend and most of the ZnO NPs dispersed in the PEG phase. The interaction of the ZnO NPs with the blend polymers slightly decreased the intensity of infrared absorption of the functional groups. The UV-blocking properties of the blends increased by 15% and 20%, and the band gap energy values were 4.1 eV and 3.8 eV for the blends loaded with ZnO NPs with a PMMA/PEG ratio of 70/30 and 30/70, respectively. In addition, the glass transition temperature (T_g_) increased by 14 °C, the crystallinity rate increased by 15%, the melting (T_m_) and crystallization(T_c_) temperatures increased by 2 °C and 14 °C, respectively, and the thermal stability increased by 25 °C compared to the PMMA/PEG blends without ZnO NP loading.

## 1. Introduction

Novel polymeric materials with improved features can be cost-effectively produced by blending different polymers [1,2,3]. However, most of the polymers are generally immiscible because the entropy of mixing is unfavorable. Therefore, when two polymers are blended, there is usually a small-scale arrangement of the phases, which is called “microstructure”. Incorporating nanoparticles (NPs) in the preparation of immiscible blends is a frequently used strategy to reduce phase separation and improve interfacial adhesion between polymer chains [4]. It also improves the thermal stability, mechanical, gas barrier, electrical conductivity and molding properties of such blends [4,5,6,7,8,9]. Immiscible polymer blends with metal NPs (e.g., polyethylene/polybutylene terephthalate/silver [10], polyethylene/polyethylene oxide/silica [11]), metal oxide (e.g., SiO_2_, ZnO and TiO_2_) NPs and carbon nanomaterials (e.g., carbon nanotubes)) are widely used in various industries, including automotive, packaging and food industries [12,13,14]. Currently, the main challenge is to optimize the polymer blend composition to minimize NP aggregation and polymer phase separation [15,16].

Polymethyl methacrylate/polyethylene glycol (PMMA/PEG) blends have been used in many applications because they are cheap and can be easily processed [17,18]. For instance, Li et al. [19] showed that compared with pure PEG, PEG melting temperature (Tm) is lower in the PEG/PMMA blend and that the mixture crystallinity progressively increases with higher PEG percentages due to the formation of spherulites. Shinzawa et al. [20] investigated the partial miscibility of PMMA/PEG blends by applying disrelation mapping to Fourier transform infrared (FTIR) spectroscopy. They concluded that PMMA/PEG blends can be partly mixed at the molecular level. Moreover, their thermal and mechanical characteristics and refractive index can be positively modulated by incorporating inorganic charges as nanofillers in the blend polymers [21,22]. Al-Hussam et al. [23] loaded a PMMA/PEG polymer blend (60 to 40 ratio) with 15 wt% LiCO_4_ NPs and various amounts of multi-walled carbon nanotubes as nanofillers. The direct current conductivity increased, whereas the glass transition temperature (T_g_) and crystallization degree decreased with the nanofiller amount increase in the polymer blend [23].

Large interfacial tensions of immiscible PMMA/PEG polymer blends lead to phase separation, and the phase-separated polymer may coalesce; this leads to larger particle size and, consequently, a deterioration of the mechanical properties [24,25,26,27,28]. In this study, the blending of PEG/PMMA was improved by adding ZnO NPs as nanofillers. Polymer blends filled with ZnO NPs were prepared by adding ZnO NPs either to PEG (dispersed phase), and then to PMMA or PMMA (minor phase), and then to the PEG matrix. The effects of ZnO NP loading and the ratio of the two orthogonal blends (7PMMA/3PEG) and (3PMMA7PEG) on the morphology of the blended polymers were investigated. In both cases, the ZnO NPs were first mixed into the minor phase (PEG or PMMA) and then incorporated into the matrix phase. As we know, the morphology of the dispersed phase (ZnO) plays an important role in the final properties of the nanocomposites. Compared with other nanofillers, ZnO NPs are easy to prepare, non-toxic [17,23,24,25,26,27], and can be used to improve the interfacial interactions between polymer blends as well as their optical, thermal, and antibacterial properties [23,24,25,26,27]. Subsequently, the obtained nanocomposites and blends were described by Fourier transform infrared spectroscopy (FTIR), scanning electron microscopy (SEM) combined with energy dispersive X-ray spectroscopy (SEM-EDX), gravimetric thermal analysis (TGA), and differential scanning calorimetry (DSC).

## 2. Experimental Section

### 2.1. Preparation of Nanocomposite Blends

About 1 g of ZnO NPs (96%, Riedel-de Haën, Seelze, Germany) was dispersed in 80 mL of ethanol (C_2_H_5_OH, 96.96%, GPR RECTAPUR, VWR, Radnor, PA, USA) with constant mixing at 50 °C for 2 h followed by sonication for 1 h to ensure ZnO NPs’ complete dispersion. PMMA/PEG/ZnO films were produced using solution casting. Two PMMA/PEG polymer blends (dry weight ratio 70/30 and 30/70) were prepared and loaded with ZnO NPs (7PMMA/3PEG/ZnO and 3PMMA/7PEG/ZnO, respectively). For solution casting, first 2 g PMMA (Vedril Spa-Resina Metallica, M_wt_ ~1.19 g/cm^3^ Motedison-Italy) was dissolved in 50 mL dichloromethane (CH_2_Cl_2_ ≥ 99.9%, Riedel-de Haën) under constant stirring at 50 °C for 15 min. Then, 2 g PEG (M_wt_ 6000 g/mol, Sigma Aldrich, St. Louis, MO, USA) was dissolved in 50 mL dichloromethane (CH_2_Cl_2_ ≥ 99.9%, Riedel-de Haën) under constant stirring at 50 °C for 15 min. When the PMMA and PEG polymers were completely dissolved and the solutions became viscous, ZnO NPs were added. For 7PMMA/3PEG/ZnO, ZnO NPs were first added to PEG and stirred at room temperature for 20 min, followed by PMMA addition. For the PEG-rich mixture (3PMMA/7PEG/ZnO), ZnO NPs were first included in the PMMA solution that was then added to PEG. Then, the PMMA/PEG/ZnO mixtures were poured on a glass substrate (10 × 10 cm) and dried at room temperature for 48 h. Table 1 lists the different samples used in this study.

### 2.2. Physiochemical Characterization

The crystallinity of ZnO NPs and polymer blends was analyzed with an X-ray diffractometer (Bruker D2 Phaser, with Cu Kα irradiation at 30 KV and 10 mA). The Debye–Scherrer equation (D = kλ/βcosθ) was used to calculate ZnO NP crystallite size. In this equation, λ represents the X-ray wavelength (1.54056 Å), θ the Bragg diffraction angle and β the full width at half maximum [29]. FTIR (JASCO-FT/IR-1400 spectrophotometer, Easton, MD, USA) characterized functional groups and bond structures. A UV-Visible-1201 spectrophotometer (Shimadzu, Kyoto, Japan) was used to determine the sample UV-Vis transmittance, and field emission SEM (Quanta 650, FEI, Hillsboro, OR, USA; accelerating voltage of 10–30 kV and low vacuum) to analyze their morphology and composition. Their melting/crystallization rate was determined by DSC (TA Instruments DSC Q20, New Castle, DE, USA) using films of 2–3 mg in weight. Heating to 180 °C was followed by cooling down to 20 °C for 15 min (heating/cooling rate of 10 °C/min). The thermal stability of the different samples was determined by TGA (Q500 instrument, TA, New Castle, DE, USA) [30]. Films of 5 mg were heated to different temperatures (30 to 700 °C; 10 °C/min) under constant nitrogen flow (60 mL/min). The measurements are based on three measurements at least.

## 3. Results and Discussion

### 3.1. ZnO NP Loading Modifies the Polymer Blend Morphology

**Figure 1:**
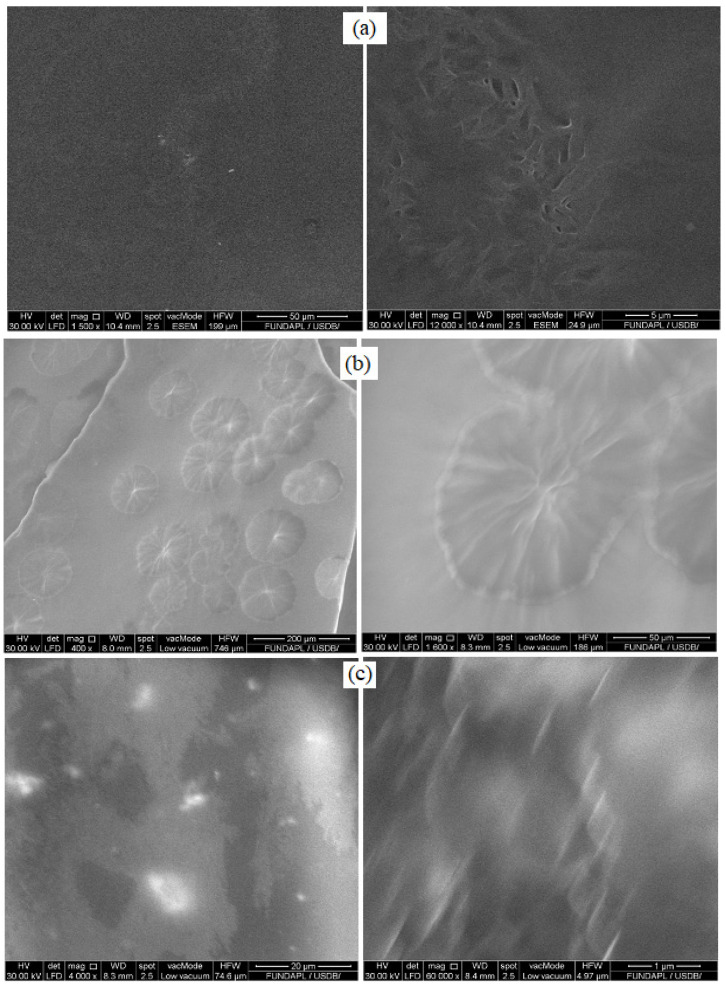

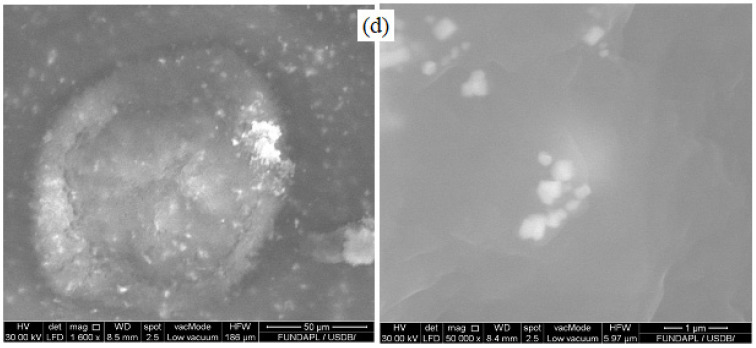
Scanning electron microscopy micrographs of the following polymer blends and nanocomposites: (**a**) 7PMMA/3PEG, (**b**) 3PMMA/7PEG, (**c**) 7PMMA/3PEG/ZnO and (**d**) 3PMMA/7PEG/ZnO.

To study the crystallization of 7PMMA/3PEG and 3PMMA/7PEG polymer blends (with/without ZnO NPs), it was important to monitor their spherulitic and crystalline morphology. PEG crystallization into spherulites was studied by SEM-EDX. The 7PMMA/3PEG blend showed PEG dispersion in the PMMA matrix like sea island, with some PEG aggregates (Figure 1a). The polymer blends without ZnO NPs displayed phase separation, especially the PEG-rich blend (Figure 1b) [31,32]. In the 3PMMA/7PEG sample, PEG had a spherulitic morphology and was dispersed in the PMMA matrix, although PMMA is a minor phase and remains the dispersing phase. This is similar to what was reported by [31], and can be explained by PEG’s high crystalline nature compared with the amorphous nature of PMMA.

**Figure 2:**
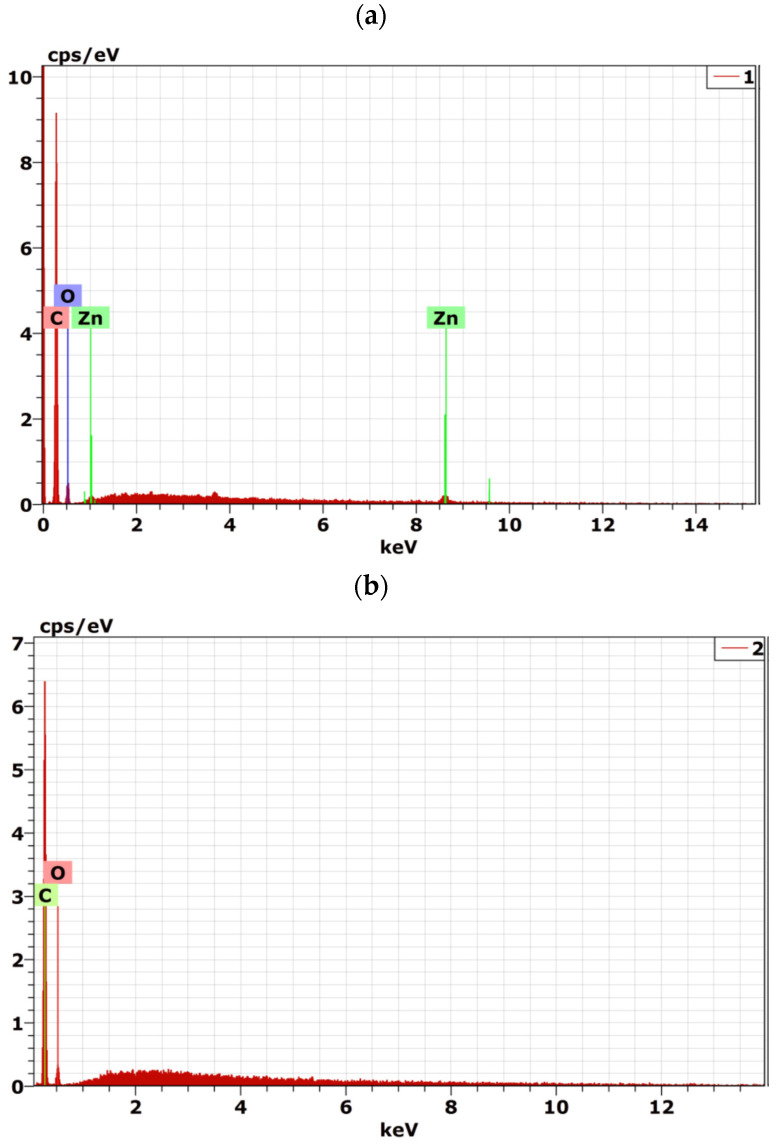
SEM-EDX spectra of (**a**) 3PMMA/7PEG/ZnO nanocomposite and (**b**) 3PMMA/7PEG blend.

Addition of ZnO NPs to the 7PMMA/3PEG blend led to a needle-like aggregate morphology of ZnO NPs with a diameter between 100 and 250 nm and an aspect ratio (l/d) of 5–10 (Figure 1c). When PEG was the major phase (3PMMA/7PEG/ZnO), ZnO NPs limited the formation of ordered PEG spherulites (Figure 1d). ZnO NPs were more likely to be dispersed in the PEG than in the PMMA phase, adding ZnO NPs decrease the PEG spherulite mean diameter by ~20% (Figure 1b,d). The developed ZnO NP morphology was sphere-like and the mean particle size was 80 nm. The different ZnO NPs morphologies in the two blends are mainly explained by how ZnO NPs were incorporated during solution preparation. For the 7PMMA/3PEG/ZnO blend, after ZnO NP addition to the dispersed PEG phase, the mixture was incorporated into the PMMA solution. For 3PMMA/7PEG/ZnO, after ZnO NP addition to the PMMA solution, the mixture was incorporated into the PEG solution. In the 7PMMA/3PEG/ZnO sample, ZnO NPs grafting to the PEG phase might have led to ZnO NP clustering into the needle-like morphology. Then, ZnO NP dispersion in the PEG/PMMA samples was investigated by SEM-EDX (Figure 2 and Table 2).

### 3.2. ZnO NP Loading Decreases the Polymer Blend Crystallinity

**Figure 3:**
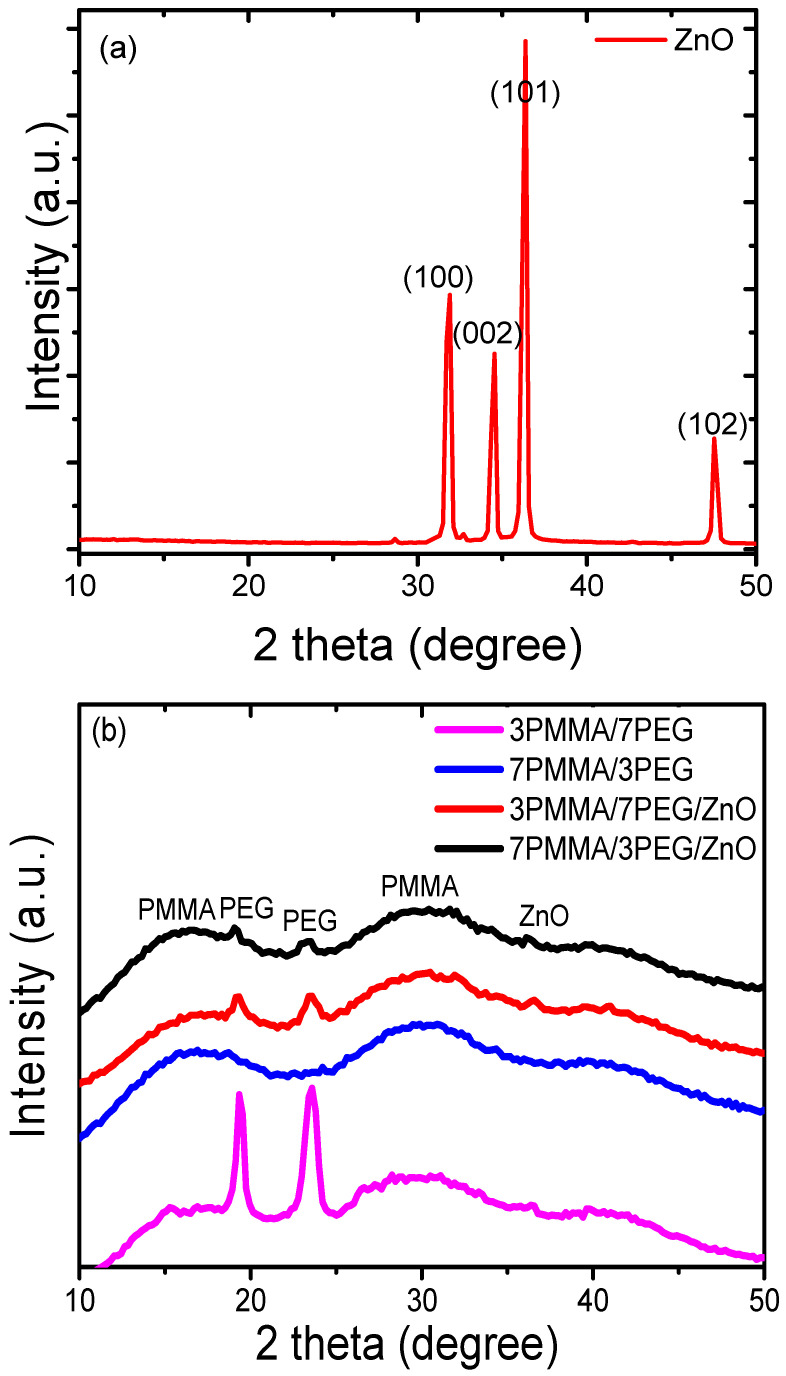
XRD diffraction profiles of: (**a**) ZnO powder, (**b**) polymer blends (7PMMA/3PEG and 3PMMA/7PEG) and nanocomposite blends (7PMMA/3PEG/ZnO and 3PMMA/7PEG/ZnO).

The XRD diagram of pure ZnO NPs showed the characteristic peaks at 31.9°, 34.5°, 36.4° and 47.5°, which corresponded to the crystal planes (100), (002), (101) and (102) and to the hexagonal wurtzite phase (JCPDS map number 36–1451) [33] (Figure 3a). Starting from the most intense diffraction peak at (101), ZnO NPs displayed a mean crystallite size of ~35 nm. The polymer blends (7PMMA/3PEG and 3PMMA/7PEG) were characterized by the presence of two diffraction peaks at 19.4° and 23.6°, which corresponded to the PEG phase crystalline part [34], and three bands at 16.9°, 30.4°, and 41.5°, which corresponded to the amorphous PMMA phase (Figure 3b) [35]. Upon ZnO NP addition, PEG peak intensity decreased significantly in the 7PMMA/3PEG/ZnO and 3PMMA/7PEG/ZnO polymer blends, mainly due to the PEG-ZnO NP interactions. ZnO NP incorporation in the blends led to the appearance of new peaks. ZnO NP addition strongly decreased the polymer blend crystallinity compared with the samples without NPs [23].

### 3.3. ZnO NP Loading Enhances the Bonding Structure of the Polymer Blends

**Figure 4:**
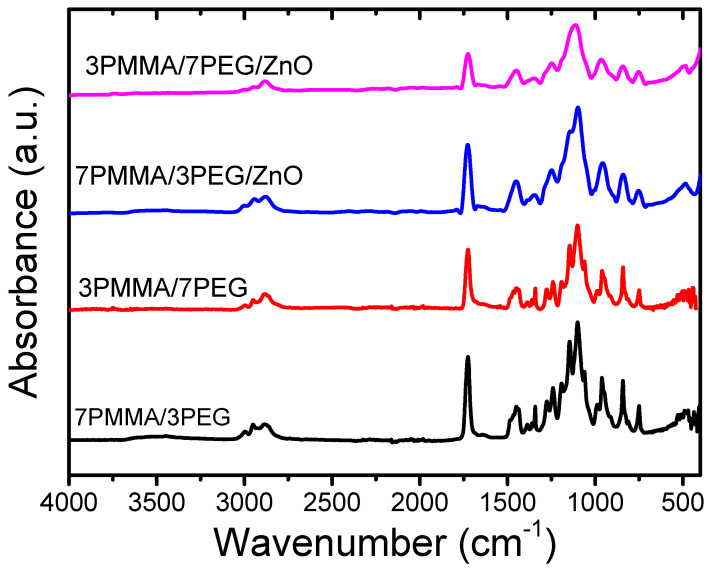
FTIR spectra of the 7PMMA/3PEG and 3PMMA/7PEG polymer blends and of the 7PMMA/3PEG/ZnO and 3PMMA/7PEG/ZnO polymer nanocomposites.

FTIR spectroscopy is a powerful tool to study possible interactions between PMMA/PEG polymer blends and ZnO NPs. The FTIR spectra of the 7PMMA/3PEG and 3PMMA/7PEG polymer blends and the 7PMMA/3PEG/ZnO and 3PMMA/7PEG/ZnO nanocomposites contained IR absorption bands at 2872, 2886 and 2867 cm^−1^ that corresponded to asymmetric and symmetric -CH_3_ stretching [36] (Figure 4). The vibrational bands at ~1725, 1722 and 1718 cm^−1^ corresponded to the C=O stretching of PMMA. The IR bands at ~1445, 1436 and 1430 cm^−1^ corresponded to the C-H deformation vibrations. The stretching C-O bands were at 1132, 1092 and 1080 cm^−1^. The bands at 970 cm^−1^, 840 cm^−1^ and 740 cm^−1^ correspond to the C-C stretching vibration, the CH_2_ vibration and the out-of-plane bending of C=O, respectively [37,38]. The vibrational band at 480 cm^−1^ in the 7PMMA/3PEG/ZnO and 3PMMA/7PEG/ZnO blends was attributed to the Zn–O bond [39]. The intensity of the IR absorption bands was higher in the 7PMMA/3PEG and 3PMMA/7PEG blends without ZnO NPs loading than with ZnO NPs loading because ZnO interacts with the polymer blend functional groups [40].

### 3.4. ZnO NP loading Improves the Polymer Blend Optical Features

**Figure 5:**
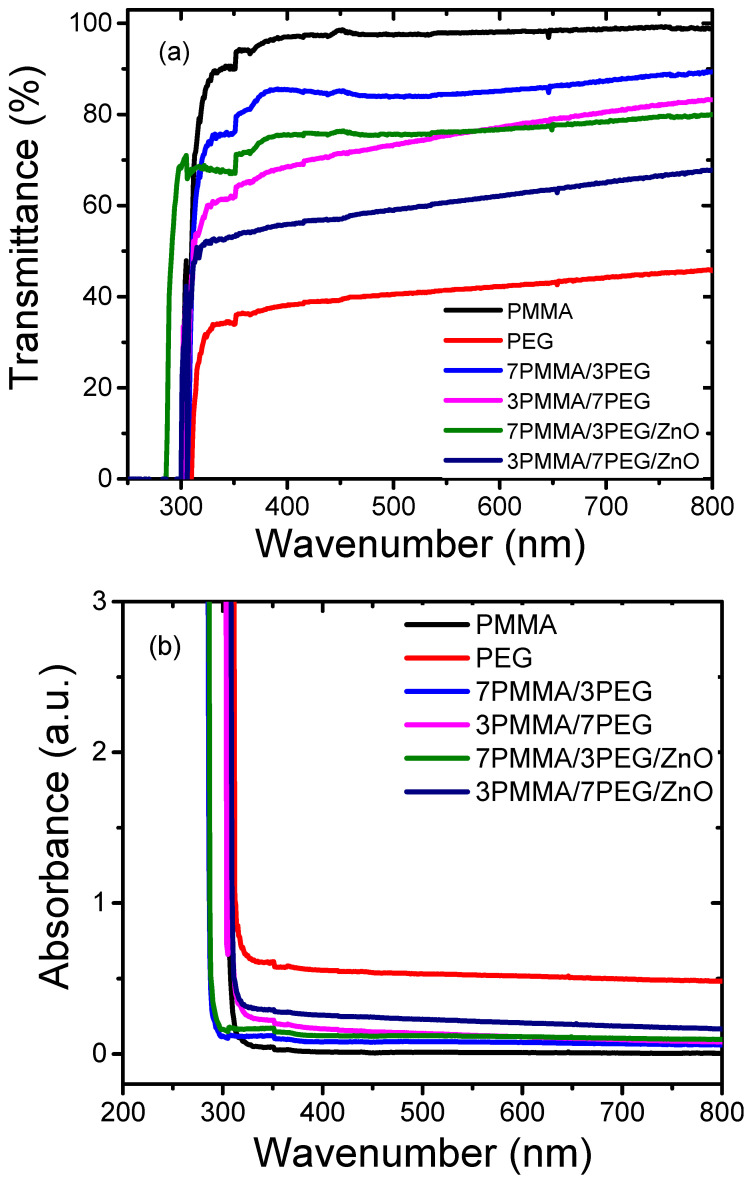
Optical properties of neat polymers (PMMA and PEG), polymer blends (7PMMA/3PEG and 3PMMA/7PEG) and polymer nanocomposite blends (7PMMA/3PEG/ZnO and 3PMMA/7PEG/ZnO). (**a**) UV-vis tranmittance (**b**) UV-vis absorbance.

Figure 5a shows the transmittance spectra of PMMA, PEG, 7PMMA/3PEG and 3PMMA/7PEG polymer blends, and 7PMMA/3PEG/ZnO and 3PMMA/7PEG/ZnO nanocomposite films. The UV transmittance of neat PMMA (95%) was higher than that of neat PEG (40%) in the UV range (200–400 nm). The PMMA spectrum showed a strong transmittance increase, with an edge at 380 nm that could be due to the n-π* transition of the carbonyl group in PMMA [41]. A comparison of the PEG and PMMA spectra indicated that PEG provides good protection in the UV range (230–400 nm). For this reason, PEG has been used in the cosmetic industry as a protection against UV radiation [42]. UV absorption was lower by the PMMA-rich blend than by the PEG-rich blend and nanocomposite (Figure 5b). In the 7PMMA/3PEG blend, PEG addition to PMMA resulted in a slight UV transmittance decrease by 8% compared with neat PMMA. Conversely, PMMA addition to PEG (7PMMA/3PEG blend) increased UV transmittance by ~40% compared with neat PEG. This could be explained by the PEG phase’s higher crystallinity degree (Figure 3b).

ZnO NP incorporation in the PMMA/PEG polymer blends decreased UV transmittance by ~15% and 20% in 7PMMA/3PEG/ZnO and 3PMMA/7PEG/ZnO polymer nanocomposites, respectively. This confirmed that the nanocomposite blends can prevent UV light transmission. The 7PMMA/3PEG/ZnO and 3PMMA/7PEG/ZnO polymer blends displayed a shoulder at 360 nm due to the chromophoric Zn–O groups [43]. These results demonstrate the interaction between ZnO NPs and PEG and PMMA polymers. Importantly, UV transmittance was lower in the PEG-rich blends (3PMMA/7PEG and 3PMMA/7PEG/ZnO) than in the PMMA-rich blends. ZnO NP inclusion in the blend increased UV light absorption by ~2.5% (7PMMA/3PEG/ZnO) and 7% (3PMMA/7PEG/ZnO) at 600 nm (Figure 5b). This could be due to the fact that ZnO NP incorporation leads to a crystallinity increase and that ZnO’s high aspect ratio in the blend increases light scattering. Shankar et al. [18] reported similar results for poly(lactide)/poly(butylene adipate-co-terephthalate)/ZnO composites and showed that the light absorption intensity was higher for the sample with than without ZnO. Similarly, ZnO NP incorporation decreased the UV-transmittance of a polylactide/PEG blend [44] and of a poly(methyl methacrylate/poly(vinylidene fluoride) blend [43].

### 3.5. ZnO NP Loading Reduces the Polymer Blend Band Gap Energy

**Figure 6:**
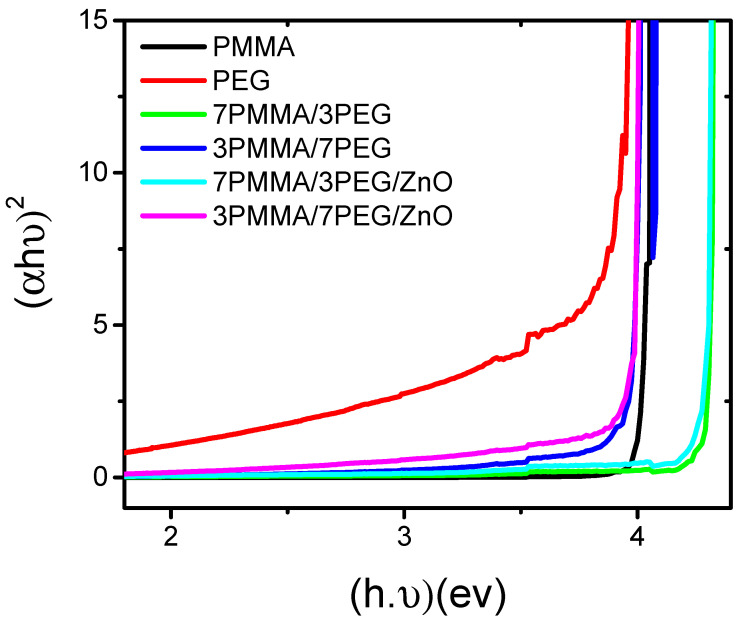
Band gap variation in function of the energy in neat polymers (PMMA and PEG), polymer blends (7PMMA/3PEG and 3PMMA/7PEG) and nanocomposite blends (7PMMA/3PEG/ZnO and 3PMMA/7PEG/ZnO).

The optical band gap is defined as the energetic distance between the filled valence and the empty conduction bands of a solid. The ability to modulate the band gap is crucial, for instance, to develop organic light-emitting diodes, sensors, electronic devices and field-effect transistors [45]. The optical band gap energy of our samples was determined using the Tauc equation [46]: ${\left(\alpha h\nu \right)}^{2}=\beta \left(\left(h\nu \right)-E_{g}\right)$, where α represents the absorption coefficient, *β* a band narrowing parameter and Eg the optical band gap. Plotting the incident photon energy (*hυ*) in the function of (*αhυ*)^2^ allowed for the determination of the Tauc diagram of the PMMA/PEG and PMMA/PEG/ZnO samples (Figure 6) [46]. ZnO NP addition in the 7PMMA/3PEG and 3PMMA/7PEG blends decreased the band gap from 4.18 to 4.11 eV and from 3.88 to 3.76 eV, respectively. This effect was mainly explained by ZnO NP incorporation into the polymer blends, as previously reported [16]. Similarly, Ramasamy et al. [47] showed that CaCO_3_ introduction in PMMA reduces the PMMA/CaCO_3_ nanocomposite band gap energy.

### 3.6. ZnO NP Loading Modulates the Polymer Blend Melting and Crystallization

**Figure 7:**
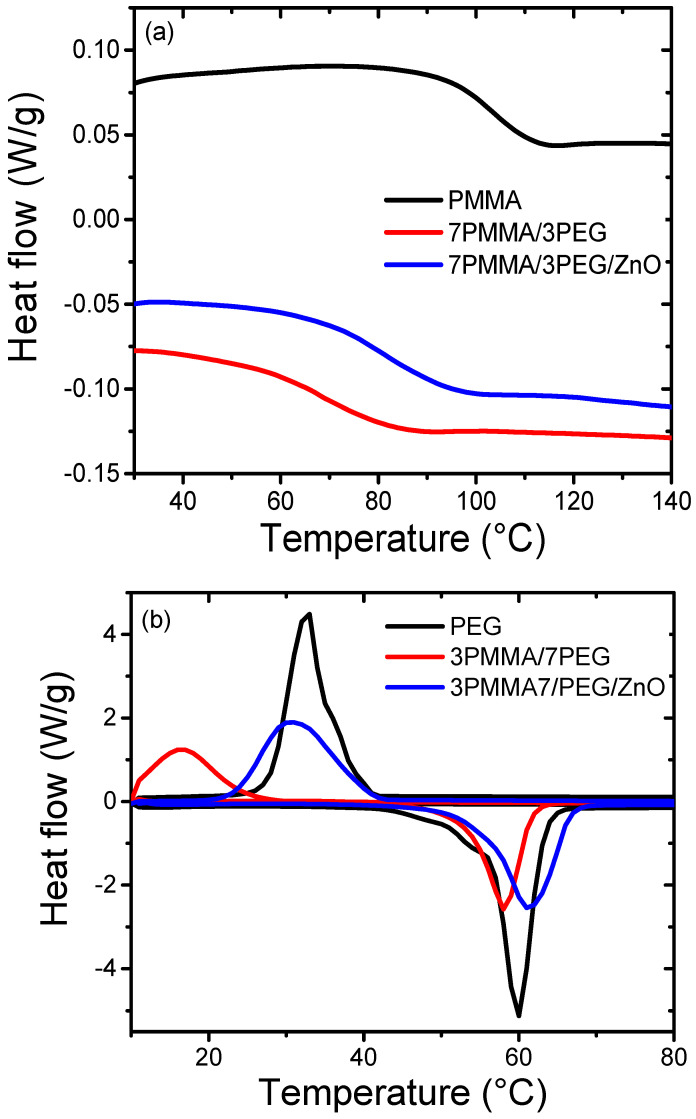
DSC curves for (**a**) neat PMMA, 7PMMA/3PEG and 7PMMA/3PEG/ZnO, and for (**b**) neat PEG, 3PMMA/7PEG and 3PMMA/7PEG/ZnO.

The thermal properties of the blends and nanocomposite blends were assessed by calculating the T_g_, crystallization temperature (T_c_), and T_m_ from DSC thermograms. Comparison of the DSC thermograms of the different samples (Figure 7) highlighted the absence of the endothermic and exothermic phases of PEG in the PMMA-rich polymer blend (7PMMA/3PEG), and, thus, zero crystallinity. Indeed, PEG is a small contributor and cannot generate a crystallization region. Compared with PMMA, the T_g_ of the 7PMMA/3PEG blend decreased by ~35° (from 102 °C to 67 °C), possibly due to PEG’s presence, which acts as a plasticizer and enhances the blend-free volume and flexibility [48]. ZnO NP incorporation into the 7PMMA/3PEG blend increased its T_g_ by ~14° (from 67 to 81 °C) compared with the 7PMMA/3PEG blend (Figure 7a). This increase could be explained by ZnO NPs’ needle-like agglomeration, which can restrict the PMMA chain mobility in the blend [23].

The thermograms of PEG-rich polymer blends (3PMMA/7PEG and 3PMMA/7PEG/ZnO) lacked the PMMA transition temperature. The T_c_ of 3PMMA/7PEG was significantly decreased by 16 °C (from 32.7 °C to 16.6 °C) compared with neat PEG. The T_c_ of 3PMMA/7PEG/ZnO was increased by 14° (from 16.6 °C to 30.5 °C) compared with the 3PMMA/7PEG blend, but remained lower than that of neat PEG. Sari et al. [32] obtained similar results in the preparation of the phase change material based on PEG/PMMA. They showed that T_c_ and T_m_ decrease by ~10° and 3°, respectively, when the amorphous phase (PMMA) concentration reaches 30% in the PEG/PMMA blend. The T_m_ of the 3PMMA/7PEG and 3PMMA/7PEG/ZnO blends was less affected (increased by 2 °C) compared with pure PEG (Figure 7b and Table 3). The crystallinity ratios of the 3PMMA/7PEG and 3PMMA/7PEG/ZnO samples decreased by ~44% and 26%, respectively, compared with the relative crystallinity of pure PEG (85%), in agreement with the XRD crystallinity data (Figure 3b). ZnO NP incorporation in the 3PMMA7PEG blend increased the 3PMMA7PEG/ZnO nanocomposite crystallinity rate from 47.7% to 62.5%. After ZnO NP addition, the crystallization enthalpy (∆H_c_) and melting enthalpy (∆H_m_) of 3PMMA/7PEG were strongly increased from 34 to 118 J/g and from 91 to 119 J/g, respectively, as previously shown by Ahmed et al. [49].

### 3.7. ZnO NP Loading Increases the Polymer Blend Thermal Stability

**Figure 8:**
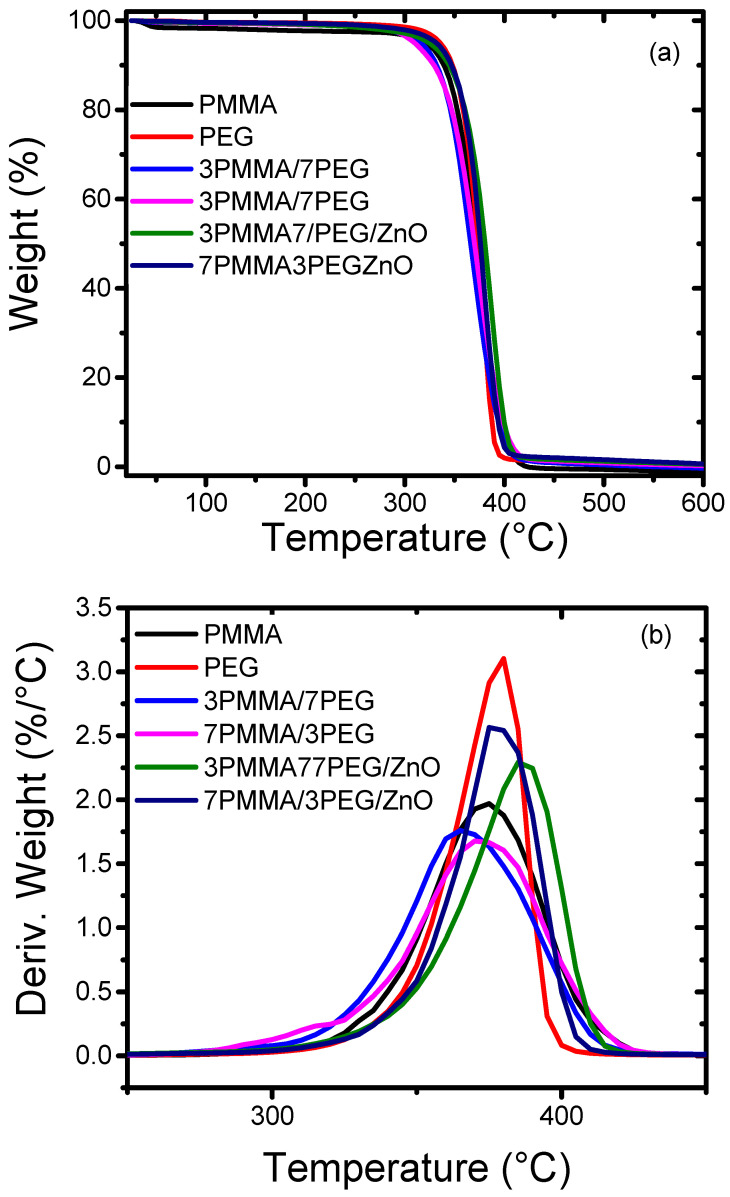
Thermogravimetry (**a**) and derivative thermogravimetry (**b**) curves of neat polymers (PMMA and PEG), polymer blends (7PMMA3PEG and 3PMMA7PEG) and nanocomposite blends (7PMMA/3PEG/ZnO and 3PMMA/7PEG/ZnO).

Then, thermal stability was investigated by thermogravimetry and derivative thermogravimetry. The thermogravimetry curves were recorded under nitrogen atmosphere (Figure 8a). Polymer blends are usually degraded in three loss stages [50]: (1) water evaporation at ~60–150 °C; (2) at ~250 °C, thermal decomposition of the functional group CO and C=O in the polymer backbones [51]; and (3) at 300–400 °C, thermal decomposition of PEG and PMMA [52]. Analysis of the decomposition temperature corresponding to 10%, 50% and maximum weight loss (T_10%_, T_50%_ and T_max_) of the samples (Table 4) showed that the T_10%_ values of the 7PMMA/3PEG and 3PMMA/7PEG/ZnO blends increased from 326° to 352 °C and from 318° to 344 °C, respectively, upon ZnO NP addition. Above 450 °C, the pure polymers and blends were completely degraded and ZnO NPs remained as char. Measurement of the char percentage indicated that ZnO NPs represented only 2% of the total mass of the nanocomposite blends, confirming the EDX results (Table 2). Comparison of the derivative thermogravimetry curves (Figure 8b) showed that ZnO NP addition to the 7PMMA/3PEG and 3PMMA7PEG blends increased the nanocomposite stability and, thus, the T_max_ increased from 359 °C to 381 °C (7PMMA/PEG/ZnO blend) and from 361 °C to 386 °C (3PMMA/7PEG/ZnO blend). This effect may be explained by the fact that the added ZnO NPs act as radical scavengers that hinder the chain cleavage and radical formation, and, thus, thermal degradation starts at higher temperatures [53].

## 4. Conclusions

One of the biggest challenges is to produce high-performance immiscible polymer blends at low cost. For industrial applications, immiscible polymers require the addition of a nanofiller to reduce production costs and improve some of their properties. However, the use of nanofiller to compatibilize immiscible polymer blends remains a challenge, requiring a high degree of control over nanofiller dispersion and localization. The composition of the polymer blends and the method by which the filler is incorporated during nanocomposite fabrication have a major impact on the quality of the nanocomposite. In this study, PMMA/PEG polymer blends with different weight ratios of PMMA and PEG (70/30 and 30/70) were prepared and ZnO NPs were added as nanofiller. The PMMA/PEG phase separation was observed in polymer blends without ZnO NPs, especially in the PEG-rich blend. Scanning electron microscopy (SEM) showed that loading ZnO NPs improved the blend miscibility, but most ZnO NPs dispersed in the PEG phase. ZnO NPs can accumulate at the polymer–polymer interface and are particularly effective in refining the microstructure of the immiscible PMMA/PEG polymer blends. The underlying mechanisms remain controversial, but two main arguments are most commonly made: compatibilizing effect and effects on interfacial rheology. UV-vis spectroscopy showed that adjusting the PMMA/PEG ratio and loading ZnO NPs improved the UV-blocking properties by 15–20%, and the band gap energy was adjusted between 4.18 eV and 3.76 eV (for 3PMMA/7PEG/ZnO). In addition, the glass transition temperature (T_g_) increased by 14 °C, the crystallinity rate increased by 15%, the melting (T_m_) and crystallization temperatures (T_c_) increased by 2 °C and 14 °C, respectively, and the thermal stability increased by 25 °C compared to the PMMA/PEG blends without ZnO NP loading.

## Figures and Tables

**Table 1:** Polymer blends and nanocomposite films used in this study.

Sample	PMMA (wt%)	PEG (wt%)	ZnO NPs (wt%)
7PMMA/3PEG	70	30	0
3PMMA/7PEG	30	70	0
7PMMA/3PEG/ZnO	70	30	5
3PMMA/7PEG/ZnO	30	70	5

ZnO NP (wt%) amount was in function of the PMMA/PEG blend total weight.

**Table 2:** Elemental composition of the polymer nanocomposites as detected by EDX.

Sample	7PMMA/3PEG		7PMMA/3PEG/ZnO	
Elements	Weight (%)	Atomic (%)	Weight (%)	Atomic (%)
Carbon (C), K	83.93	87.43	82.37	87.18
Oxygen (O), K	16.07	12.57	15.66	0.38
Zinc (Zn)	-	-	1.98	12.44
Total	100	100	100	100

K: Spectroscopic term related to EDX.

**Table 3:** DSC results of the neat polymers, polymer blends and nanocomposites.

Samples	Second and Third Heating Scan					
	T_g_ (°C)	T_c_ (°C)	T_m_ (°C)	∆H_c_ (J/g)	∆H_m_ (J/g)	Crystallization Rate (%)
PMMA	102 ± 1	--	--	--	--	--
PEG	--	32 ± 2	59 ± 2	157 ± 3	162 ± 3	85 ± 3
7PMMA/3PEG	67 ± 2	--	--	--	--	--
3PMMA/7PEG	--	16 ± 3	58 ± 1	34 ± 2	91 ± 2	48 ± 4
7PMMA/3PEG/ZnO	81 ± 1	--	--	--	--	--
3PMMA/7PEG/ZnO	--	30 ± 1	60 ± 2	118 ± 3	119 ± 1	63 ± 2

**Table 4:** Thermal stability of neat polymers, blends and nanocomposites by thermogravimetry.

Samples	PMMA	PEG	7PMMA/3PEG	3PMMA/7PEG	7PMMA/3PEG/ZnO	3PMMA/7PEG/ZnO
T_10%_ (°C)	338 ± 3	352 ± 2	326 ± 3	318 ± 4	352 ± 3	344 ± 2
T_50%_ (°C)	371 ± 2	380 ± 3	359 ± 4	361 ± 4	381 ± 2	373 ± 3
T_max_ (°C)	374 ± 1	380 ± 3	359 ± 3	361 ± 2	381 ± 1	386 ± 2
Residue (%)	0.10 ± 0.01	0.39 ± 0.02	0.15 ± 0.02	0.52 ± 0.03	2.2 ± 0.05	1.88 ± 0.02

## Data Availability

The authors confirm that the data supporting the findings of this study are available within the article.
